# Yap1-mediated Flr1 expression reveals crosstalk between oxidative stress signaling and caffeine resistance in *Saccharomyces cerevisiae*

**DOI:** 10.3389/fmicb.2022.1026780

**Published:** 2022-11-23

**Authors:** Ji Eun Choi, Seo-Hee Heo, Woo-Hyun Chung

**Affiliations:** ^1^College of Pharmacy, Duksung Women’s University, Seoul, South Korea; ^2^Innovative Drug Center, Duksung Women’s University, Seoul, South Korea

**Keywords:** oxidative stress response, caffeine resistance, Yap1, Flr1, DNA damage, adaptation, *Saccharomyces cerevisiae*

## Abstract

Caffeine, a methylxanthine derivative, affects various physiological conditions such as cell growth, proliferation, and energy metabolism. A genome-wide screening for genes required for caffeine resistance in *Schizosaccharomyces pombe* revealed several candidates, including Pap1 and downstream target genes involved in caffeine efflux. We found that Yap1, a budding yeast AP-1 homolog required for oxidative stress response, has a caffeine tolerance function. Although the *Yap1* mutant is not sensitive to caffeine, overexpression of Yap1 renders cells resistant to high concentrations of caffeine. Caffeine sensitivity of mutants lacking two multidrug transporters, Pdr5 or Snq2, is completely recovered by Yap1 overexpression. Among Yap1-dependent target genes, *FLR1*, a fluconazole-resistant gene, is necessary but not sufficient for caffeine tolerance. Low concentrations of hydrogen peroxide induce Yap1 activation, which restores cell viability against caffeine toxicity. Intriguingly, oxidative stress-mediated cellular adaptation to caffeine toxicity requires Yap1, but not Flr1. Moreover, caffeine is involved in reduction of intracellular reactive oxygen species (ROS), as well as mutation rate and Rad52 foci formation. Altogether, we identified novel reciprocal crosstalk between ROS signaling and caffeine resistance.

## Introduction

Caffeine is a natural purine analog and one of the most frequently ingested methylxanthine derivatives in beverages and medications. It has a wide variety of biological effects with multiple cellular targets in fungi, plants, and humans. In higher animals, caffeine plays beneficial roles in neuronal stimulation accompanied by antidepressant and diuretic effects (López-Cruz et al., 2018). Caffeine is a relatively non-selective drug that affects multiple cellular processes such as cell growth, proliferation, and energy metabolism by triggering calcium mobilization and inhibiting the target of rapamycin (TOR) signaling as well as the DNA damage repair system (Combettes et al., 1994; Ruta and Farcasanu, 2020).

Several studies have reported that in yeast, caffeine threatens cell wall integrity and causes cytotoxicity by perturbing several steps in DNA damage repair pathways and cell cycle checkpoint signaling (Martin et al., 2000; Moser et al., 2000; Kuranda et al., 2006). Caffeine inhibits the repair of UV-induced pyrimidine dimers by interfering with DNA photolyase and UvrA. It also disrupts the recruitment of Sae2 and Dna2 nucleases required for break end resection, one of the early steps in homologous recombination (HR), and Rad51, a strand exchange protein that detects repair homology, leading to failure in DNA double-strand break (DSB) repair (Selby and Sancar, 1990; Zelensky et al., 2013; Tsabar et al., 2015a, 2015b).

A genome-scale screen for caffeine-sensitive mutants in the fission yeast *Schizosaccharomyces pombe* has revealed that mutants lacking *RAD3*, *RAD51*, *RAD54*, and *SSB3*, which are responsible for HR and damage checkpoint activation, are all highly sensitive to caffeine (Calvo et al., 2009). Caffeine disturbs cell cycle progression and exerts mutagenic effects through the suppression of Tel1 and Mec1, yeast homologs of ataxia-telangiectasia mutated (ATM) and ATM-related (ATR) kinases for checkpoint signaling (Saiardi et al., 2005).

Interestingly in *S. pombe*, caffeine sensitivity and toxicity are also involved in the level of intracellular reactive oxygen species (ROS) and the ensuing activation of the oxidative stress response. Significant caffeine tolerance has been observed in mutants with increased activity of Pap1, an AP-1-like transcription factor in *S. pombe*, that induces the expression of various antioxidant enzymes against oxidative stress (Benko et al., 2004). Cells lacking Pap1 display a highly sensitive phenotype to caffeine, and in this case, caffeine resistance is largely due to Hba2, a Pap1-mediated drug efflux pump (Calvo et al., 2009). The lack of Trr1, a thioredoxin reductase, causes an elevation of intracellular ROS leading to constitutive activation of Pap1 and caffeine tolerance (Vivancos et al., 2004; Calvo et al., 2009). These observations indicate that cellular responses to caffeine might be linked to the oxidative stress response pathway or the ROS signaling process (Chung, 2021).

Yap1, a Pap1 homolog in *Saccharomyces cerevisiae*, is crucial for the oxidative stress response (Rodrigues-Pousada et al., 2019). Yap1 shuttles between the cytoplasm and the nucleus mediated by Crm1, an exportin, and by Pse1, an importin (Yan et al., 1998; Isoyama et al., 2001). Unlike *S. pombe* counterparts that are dispensable for survival, both Crm1 and Pse1 are essential in *S. cerevisiae*, indicating that the localization or cellular distribution of Yap1 is critical to maintaining cellular homeostasis. Overexpression of Yap1 renders cells resistant to several toxic chemicals, such as diazaborine and cadmium, mediated by the transcriptional induction of Flr1 and Ycf1, two multidrug transporters, in a *Yap1*-dependent manner (Wemmie et al., 1994; Jungwirth et al., 2000).

In this study, we assessed whether the oxidative stress pathway is involved in caffeine tolerance in *S. cerevisiae*. Strong caffeine resistance was observed when Yap1 is overexpressed, whereas the lack of Yap1 made no difference in caffeine sensitivity. We discovered that Flr1, a fluconazole-resistant transporter, is mainly responsible for caffeine tolerance under Yap1 activation. While intracellular localization and/or activation of Yap1 is not affected by caffeine, Yap1-mediated oxidative stress response confers resistance to caffeine toxicity. Based on the results that caffeine reduces the level of intracellular ROS, spontaneous mutation frequency, as well as DNA damage-induced foci formation, we suggest a significant reciprocal crosstalk between ROS signaling and caffeine resistance, which provides a cooperative mechanism for the maintenance of cell integrity.

## Materials and methods

### Strains, plasmids, and growth media

All yeast strains used in this study are isogenic derivatives of *S. cerevisiae* BY4741 (*MAT***a** *his3*Δ1 *leu2*Δ0 *lys2*Δ0 *ura3*Δ0) obtained from Yeast Knockout (YKO) collection (YSC1053 glycerol stock, Thermo Fisher Scientific). The genotypes of all strains used in this study are listed in Supplementary Table 1. The strains with C-terminally GFP-fused proteins, Yap1-GFP and Rad52-GFP, were constructed by oligonucleotide-directed in-frame tagging method as previously described (Huh et al., 2003). Yeast cell cultures and treatments with caffeine, hydrogen peroxide (H_2_O_2_) or phleomycin were performed in YEPD (1% yeast extract, 2% peptone, and 2% dextrose) or minimal SD media supplemented with the required amino acids. Overexpression plasmids including *YAP1*, *SKN7*, *SOD1*, *TSA1*, or *FLR1* were created using high copy number shuttle vector pRS426 with the wild-type *ADH1* promoter (Mumberg et al., 1995).

### Drug sensitivity analysis

The sensitivity of yeast cells to drugs was determined by spot plating assay. A small part of the overnight-grown cell culture was taken and grown again in fresh liquid media to reach mid-log phase (3–4 × 10^7^ cells/ml). Cells were then diluted 10-fold serially and spotted in rows onto YEPD or synthetic dropout (SD) plates containing H_2_O_2_ and/or caffeine. After spotting, cells were incubated for 3 days at 30°C and then photographed.

### Fluorescence microscopy

Fluorescence microscopic pictures were acquired with Leica DMi8 automated microsystems. LAS X software was used for the image analysis. To detect localization of Yap1-GFP to the nucleus, green fluorescent cells merged with DAPI stain signals were quantified after H_2_O_2_ or caffeine treatment. To measure the amount of DSB lesions and genomic instability, the percentage of cells with subnuclear Rad52-GFP foci formation in the population was determined (Lisby et al., 2001). At least 300 individual cells for each experiment were counted and analyzed manually.

### RNA isolation and reverse transcription-PCR (RT-PCR) analysis

Total RNA was extracted using Trizol reagent (Invitrogen) according to the manufacturer’s instructions. The quality of the isolated RNA was assessed by Agilent 2,100 bioanalyzer using the RNA 6000 Nano Chip (Agilent Technologies). The concentration of RNA was determined by measuring the UV absorbance at 260 nm in a spectrophotometer, and highly purified samples with comparable A260/A280 ratios (1.8 ~ 2.1) were used for RT-PCR analysis. The sequences of the PCR primer pairs for the amplification of target genes were shown in Supplementary Table 2. PCR was performed with T100 thermal cycler (BIO-RAD). All amplification set-up went through 22 ~ 28 cycles unless noted otherwise.

### The QuantSeq mRNA sequencing and data analysis

The 3’ mRNA-Seq libraries were prepared using QuantSeq 3’ mRNA-Seq Library Prep Kit (Lexogen) following the manufacturer’s instructions. Each 500 ng total RNA was hybridized to the oligo-dT primer containing an Illumina-compatible sequence at its 5′ end. After reverse-transcription and degradation of the RNA template, second strand synthesis was initiated by a random primer containing an Illumina-compatible linker sequence at its 5′ end and was purified. The library was amplified to add the complete adapter sequences required for cluster generation. High-throughput sequencing was determined using the single-end 75 sequencing platform of NextSeq 500 (Illumina). QuantSeq 3′ sequencing reads were aligned using Bowtie2 software (Langmead and Salzberg, 2012). Differential gene expression was evaluated based on counts from unique and multiple alignments using coverage in Bedtools and was determined using EdgeR within R (R development Core Team, 2016). Gene classification was based on searches done by DAVID^1^ and Medline databases.^2^ Student’s *t*-test was used to determine significant differences (value of *p* < 0.05). All of raw sequencing data is deposited at NCBI GEO site.^3^

### Measurement of intracellular ROS level

To measure intracellular ROS level in yeast, cells were grown to exponential phase and diluted to an OD_600_ of ~0.2. H_2_DCFDA (2′,7′-dichlorodihydrofluorescein diacetate, Thermo Fisher Scientific), a cell-permeant indicator for ROS detection, was added at a final concentration of 5 μg/ml, and then cells were incubated with shaking for 2 h at 30°C. Intracellular ROS levels were determined using a BD FACS Canto II flow cytometer (Becton Dickinson) as previously described (Madeo et al., 1999). A baseline of zero (background level of fluorescence) was set based on the maximum value of control sample without the ROS indicator. The cells with higher ROS level than background were counted and converted into a percentage. All values represent the average of at least three independent experiments.

### Measurement of cell viability and mutation frequency

Yeast cells were inoculated into 5 ml of YEPD media and grown overnight at 30°C. The next day, cells were diluted into 5 ml of fresh media to an OD_600_ of ~0.2 and incubated with shaking for 6 h. More than 100 cells were counted and plated onto three YEPD plates including H_2_O_2_ and/or caffeine at the indicated final concentrations. The plates were incubated at 30°C for 3 days and then colonies were counted for a colony forming unit (CFU) assay. The rate of spontaneous or induced mutations as a result of genome instability was measured by a forward-mutation assay that detects mutations in the *CAN1* gene (Motegi and Myung, 2007). Yeast cells were treated with H_2_O_2_ and/or caffeine for 2 h at the indicated concentrations and then were grown on plates with or without 60 μg/ml canavanine. Spontaneous mutation rates were determined by counting CFUs after incubation for 3–4 days at 30°C. All rates represent the average of three independent experiments and error bars indicate the standard deviation.

## Results

### Cells lacking Yap1 are not sensitive to caffeine whereas overexpression of Yap1 confers resistance

The screening analysis of several caffeine-resistant mutants using fission yeast *S. pombe* strains has revealed that all the caffeine-tolerant phenotypes identified are related to an elevated expression of Pap1 or its constitutive localization in the nucleus (Kumada et al., 1996; Benko et al., 2004). In *S. pombe*, there are two alternative oxidative stress responses by which different signaling pathways are triggered. One is mediated by Pap1, an AP-1-like transcription factor, responding to moderate concentrations of H_2_O_2_, and the other is the Sty1 MAP kinase pathway activated not only by oxidative stress but also by general stress conditions such as heat shock and osmotic stress (Toone et al., 1998; Vivancos et al., 2004). Overexpression of Pap1, but not Sty1, makes cells resistant to high doses of caffeine, and Pap1- and Sty1-deficient cells are both highly sensitive to caffeine (Benko et al., 2004; Calvo et al., 2009).

**Figure 1:**
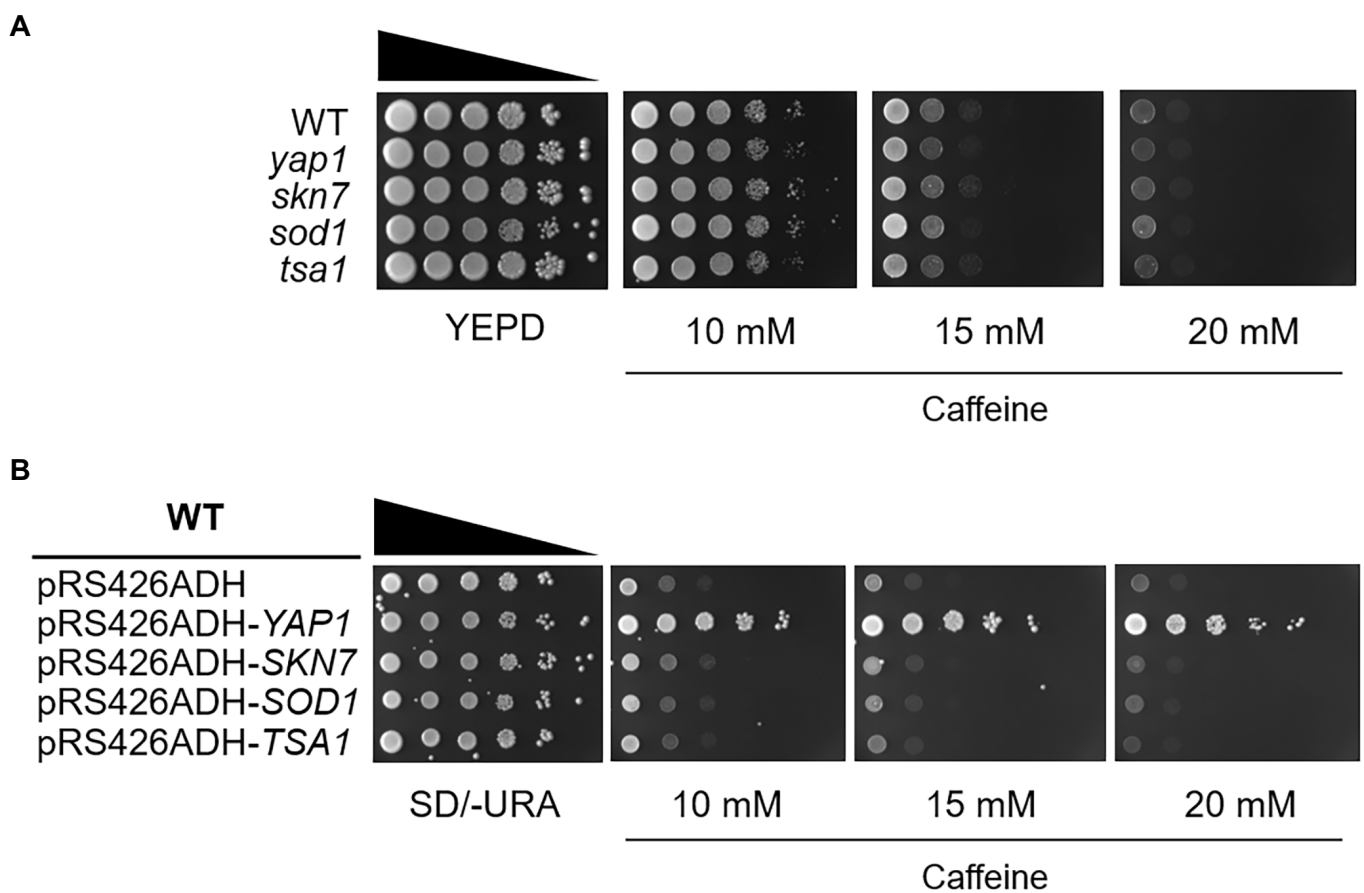
The *yap1* null mutant is not sensitive to caffeine while overexpression of *Yap1* confers significant resistance. **(A)** 10-fold serial dilutions of yeast mutant strains were spotted on YEPD media in the presence of caffeine and incubated for 3 days at 30°C. **(B)** 10-fold serial dilutions of yeast overexpression strains were spotted on SD/-URA media containing caffeine and incubated for 3 days at 30°C.

Based on these observations, we examined the caffeine sensitivity of cells lacking Yap1 or Skn7, two specialized transcriptional regulators for oxidative stress response in *S. cerevisiae*. Skn7 collaborates with Yap1 on several promoters such as *TRX2* and *AHP1* to induce transcription in response to oxidative stress (Morgan et al., 1997; Lee et al., 1999). In contrast with the homologs in *S. pombe*, we could not observe any caffeine sensitivity in *yap1* or *skn7* null mutants (Figure 1A). The expression of Sod1 and Tsa1, two representative antioxidant enzymes, is mainly under the control of Yap1 and/or Skn7. When caffeine sensitivity was examined in cells lacking Sod1 or Tsa1, no difference in sensitivity was shown. Next we overexpressed *YAP1*, *SKN7*, *SOD1,* or *TSA1* in WT yeast cells using the multicopy plasmid pRS426ADH, and found that only the expression of Yap1 confers yeast cells with significantly enhanced resistance to high doses of caffeine (Figure 1B).

### Caffeine sensitivity of the *flr1* null mutant is not overcome by Yap1 overexpression

**Figure 2:**
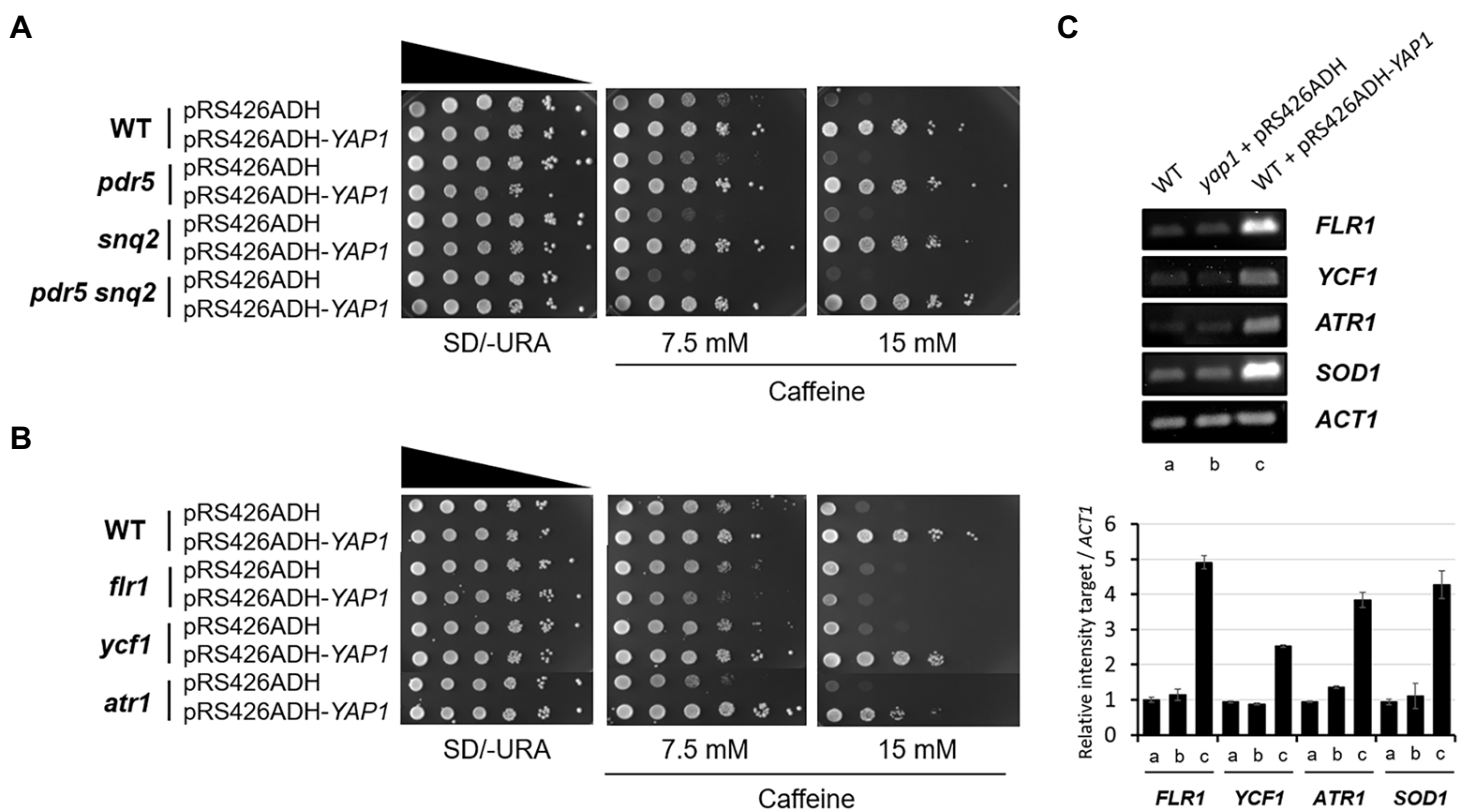
Caffeine sensitivity of *flr1* mutant is not recovered by *Yap1* overexpression. **(A,B)** 10-fold serial dilutions of yeast mutant strains with *YAP1* overexpression plasmids were spotted on SD/-URA media containing caffeine and incubated for 3 days at 30°C. **(C)** RT-PCR analysis of *Yap1*-dependent target genes expressed in WT, *yap1* and *YAP1* overexpression strains. *ACT1* as a loading control. The products were analyzed by electrophoresis on a 1.5% agarose gel. The numbers of PCR cycles used are 22 (*FLR1* and *SOD1*), 28 (*YCF1* and *ATR1*) and 30 (*ACT1*), respectively.

Previously, in multicopy vector-based genomic library screening, two ATP-binding cassette (ABC) transporters, Pdr5, and Snq2, have been identified as major caffeine efflux pumps in *S. cerevisiae* (Kolaczkowski et al., 1996; Tsujimoto et al., 2015). We evaluated whether caffeine tolerance mediated by Yap1 is involved in the function of these multidrug resistance transporters. Consistent with the previous report that Snq2 is more functional in caffeine efflux than Pdr5, the *snq2* mutant was slightly more sensitive to caffeine than the *pdr5* mutant, and the *pdr5 snq2* double mutant showed even more sensitivity than each of the single mutants (Figure 2A). However, we observed that the overexpression of Yap1 almost completely surmounts caffeine sensitivity in the *pdr5 snq2* double mutant as well as each of the single mutants. These results suggest that caffeine tolerance is associated with elevated levels of Yap1 and is not derived from an improvement in caffeine efflux mediated by either Pdr5 or Snq2 (Figure 2A).

Flr1, Ycf1 and Atr1, three efflux pumps in *S. cerevisiae*, confer resistance to several drugs under the control of Yap1. Ycf1 is required for cadmium tolerance and arsenite detoxification (Wemmie et al., 1994; Ghosh et al., 1999). Flr1 is essential for Yap1-mediated resistance to fluconazole, cycloheximide, aminotriazole, and 4-nitroquinoline-N-oxide (4-NQO; Alarco et al., 1997), and both Ycf1 and Flr1 are involved in Yap1-mediated defense against cerulenin and diazaborine toxicity (Oskouian and Saba, 1999; Jungwirth et al., 2000). In addition, Atr1 is responsible for resistance to 4-NQO in both Yap1- and Gcn4-dependent manners (Coleman et al., 1997).

To examine whether these membrane-associated transporters are involved in the acquisition of caffeine resistance in a Yap1-related manner, each deletion mutant was spotted onto caffeine-infused plates with or without Yap1 overexpression. Except for a slightly sensitive *atr1* mutant, neither the *flr1* nor the *ycf1* mutant was found to be sensitive to caffeine compared to the WT strain under normal conditions (Figure 2B). Interestingly, however, caffeine resistance of the *flr1* mutant is not enhanced by Yap1 overexpression at all. The caffeine resistance of *ycr1* and *atr1* mutants is just slightly less than that of WT strain. These results suggest that *FLR1* is a major target gene regulated by Yap1 and is responsible for a specific Yap1-dependent caffeine tolerance. Analysis with reverse transcription-PCR (RT-PCR) revealed that the expression level of Flr1 is not almost altered in *yap1* mutants compared to WT and increases significantly in cells that overexpress Yap1 (Figure 2C). When Yap1 is overexpressed, the amount of transcriptional induction in *FLR1* is higher than that of *YCF1* or *ATR1*. This accounts for our previous results that there is no difference in caffeine sensitivity between WT and the *yap1* mutant but Yap1 overexpression increases resistance (Figure 1).

### Flr1 is required but not sufficient for the increase of Yap1-dependent caffeine tolerance

**Figure 3:**
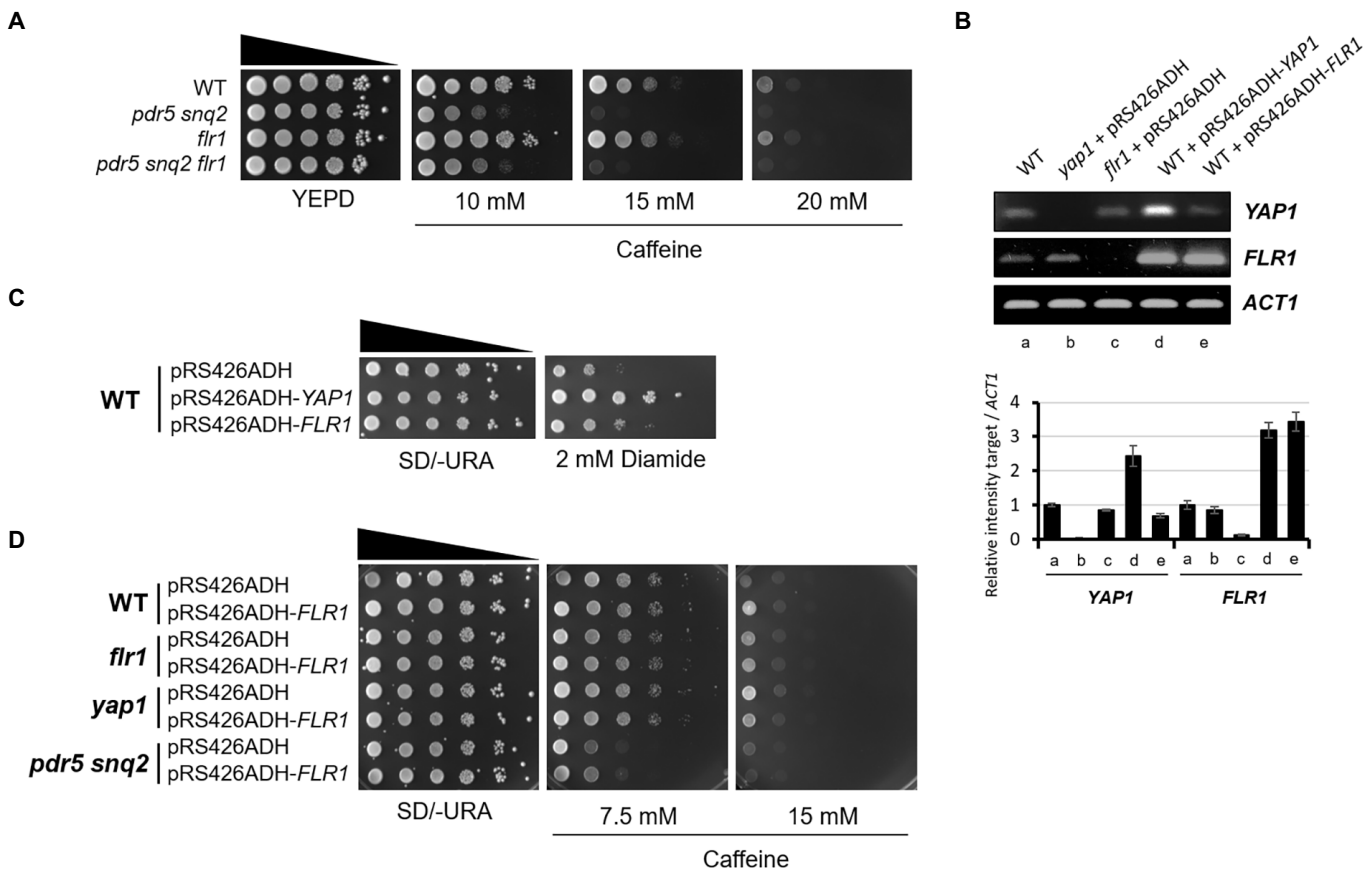
Flr1 is required but not sufficient for the increase of Yap1-dependent caffeine tolerance. **(A)** 10-fold serial dilutions of yeast mutant strains were spotted on YEPD media containing caffeine and incubated for 3 days at 30°C. **(B)** RT-PCR analysis of *YAP1* and *FLR1* expressed in WT, *yap1*, *flr1*, and strains overexpressing *YAP1* or *FLR1*. *ACT1* as a loading control. The numbers of PCR cycles used are 28 (*YAP1*), 22 (*FLR1*) and 25 (*ACT1*), respectively. **(C)** 10-fold serial dilution of yeast strains with *YAP1* or *FLR1* overexpression were spotted on SD/-URA media containing diamide and incubated for 3 days at 30°C. **(D)** 10-fold serial dilutions of yeast mutant strains with *FLR1* overexpression plasmids were spotted on SD/-URA media containing caffeine and incubated for 3 days at 30°C.

Contrary to mutant cells carrying deletions in both *PDR5* and *SNQ2* that have considerable caffeine sensitivity, the *flr1* mutant is as sensitive to caffeine as the WT strain, and the *pdr5 snq2 flr1* triple mutant is as sensitive as the *pdr5 snq2* double mutant. This suggests that the Flr1 transporter alone is not significantly involved in caffeine efflux when compared to Pdr5 and Snq2 under no Yap1 overexpression conditions (Figure 3A).

To investigate whether Flr1 could functionally substitute Pdr5 or Snq2 in caffeine export, we overexpressed *FLR1* in a *pdr5 snq2* double mutant and tested its caffeine resistance. *FLR1* was overexpressed under the control of the *ADH1* promoter and its transcription level was compatible with that of Yap1 overexpression strain (Figure 3B). Consistent with the previous report by Nguyên et al. (2001), the amount of Flr1 produced was also enough to show resistance to diamide, a thiol oxidizing agent (Figure 3C). However, we found that the caffeine sensitivity of the *pdr5 snq2* double mutant was not sufficiently recovered by Flr1 overexpression, indicating that Flr1 is not functionally redundant with Pdr5 or Snq2 in caffeine tolerance (Figure 3D). This also implies that Flr1 alone is not enough to endure a caffeine threat or to show strong caffeine resistance and cells still need to employ more unknown target(s) mediated by activated Yap1 besides the Flr1 transporter.

### Neither Yap1 activation nor Flr1 expression is induced by caffeine

**Figure 4:**
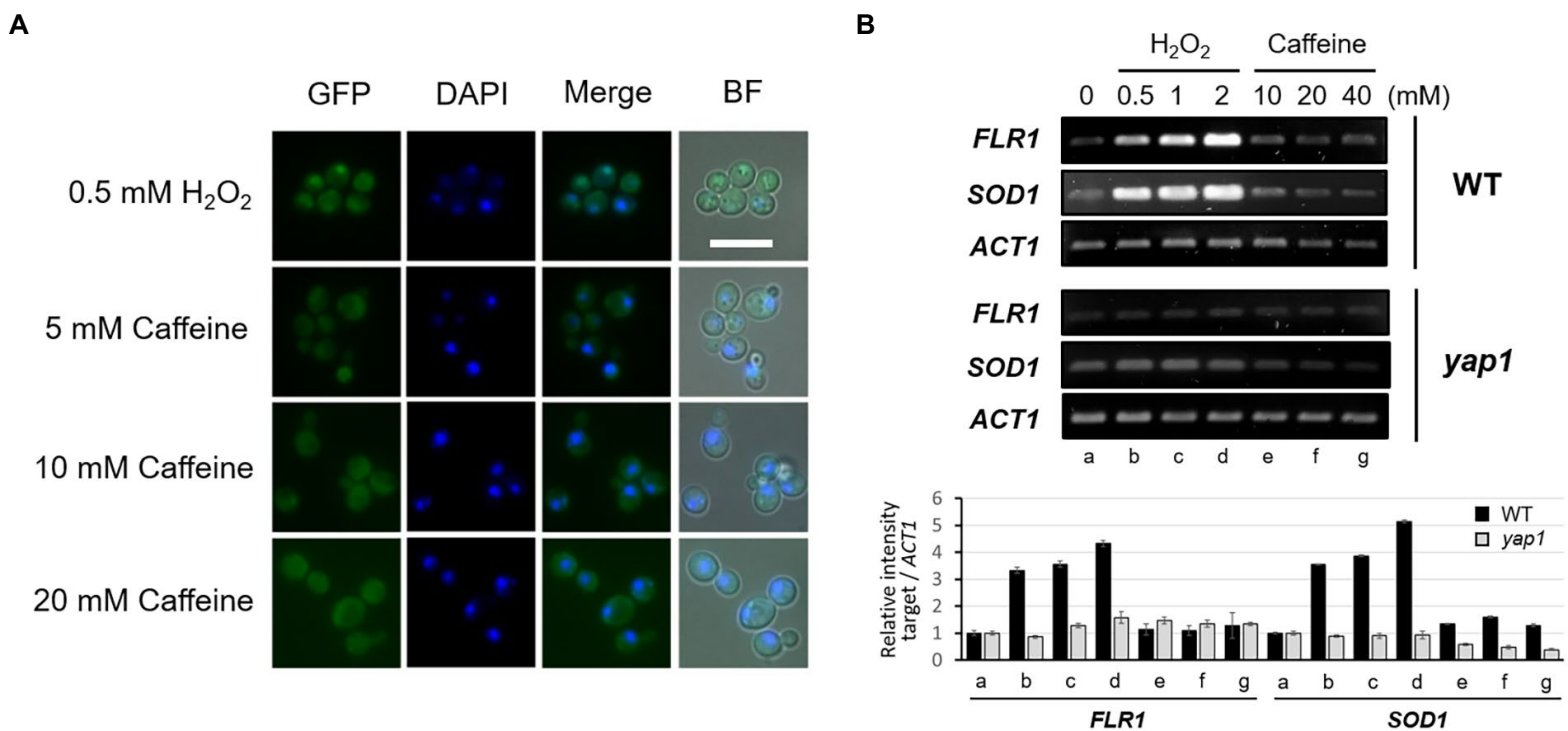
Activation of Yap1 and its intracellular localization are not affected by caffeine treatment. **(A)** Fluorescent microscopy of the spontaneous or induced nuclear localization of Yap1-GFP in WT with H_2_O_2_ or caffeine treatment. White scale bar corresponds to 10 μm. **(B)** RT-PCR analysis of *FLR1* and *SOD1* expression in WT and *yap1* mutant. *ACT1* as a loading control. Cells were incubated at 30°C for 30 min with H_2_O_2_ or caffeine treatment. The numbers of PCR cycles used are 25 (*SOD1*) and 28 (*FLR1* and *ACT1*), respectively.

Previous studies have shown that Yap1 is activated and accumulates in the nucleus specifically under oxidative stress conditions and its absence causes hypersensitivity to several oxidants such as hydroperoxides and diamide (Kuge and Jones, 1994; Rodrigues-Pousada et al., 2019). We examined whether Yap1 and the transcription of its downstream target genes are activated by caffeine. Yap1 is activated and is relocated to the nucleus within 30 min of hydrogen peroxide treatment, whereas it is largely cytoplasmic with no changes after caffeine treatment even at high concentrations (Figure 4A).

We next performed RT-PCR analysis to inquire about a possible alteration in *FLR1* expression level after H_2_O_2_ or caffeine treatment. As shown in Figure 4B, transcription of *FLR1* is induced by H_2_O_2_, but not by caffeine, only in the presence of functional Yap1, and its induction pattern is quite similar to that of *SOD1*, another target of Yap1 induction. Caffeine tolerance rendered by Yap1 overexpression in the presence of Flr1 was induced by the oxidative stress response, but not by caffeine itself. These results imply that intracellular caffeine response and ROS signaling may have a cooperative linkage to maintain cell integrity.

### Flr1 expression is regulated mainly by Yap1 and is important for caffeine tolerance

Caffeine may alter the transcriptional profiles of multiple genes that are responsible for the transport of various metabolites and proteins. Kuranda et al. (2006) reported that the expression of at least 50 genes implicated in cellular transport was elicited by 20 mM caffeine treatment. DNA microarray studies have revealed that the expression of three multidrug transporter genes, *SNQ2*, *QDR3,* and *MDL2*, was upregulated by caffeine (Kuranda et al., 2006).

**Figure 5:**
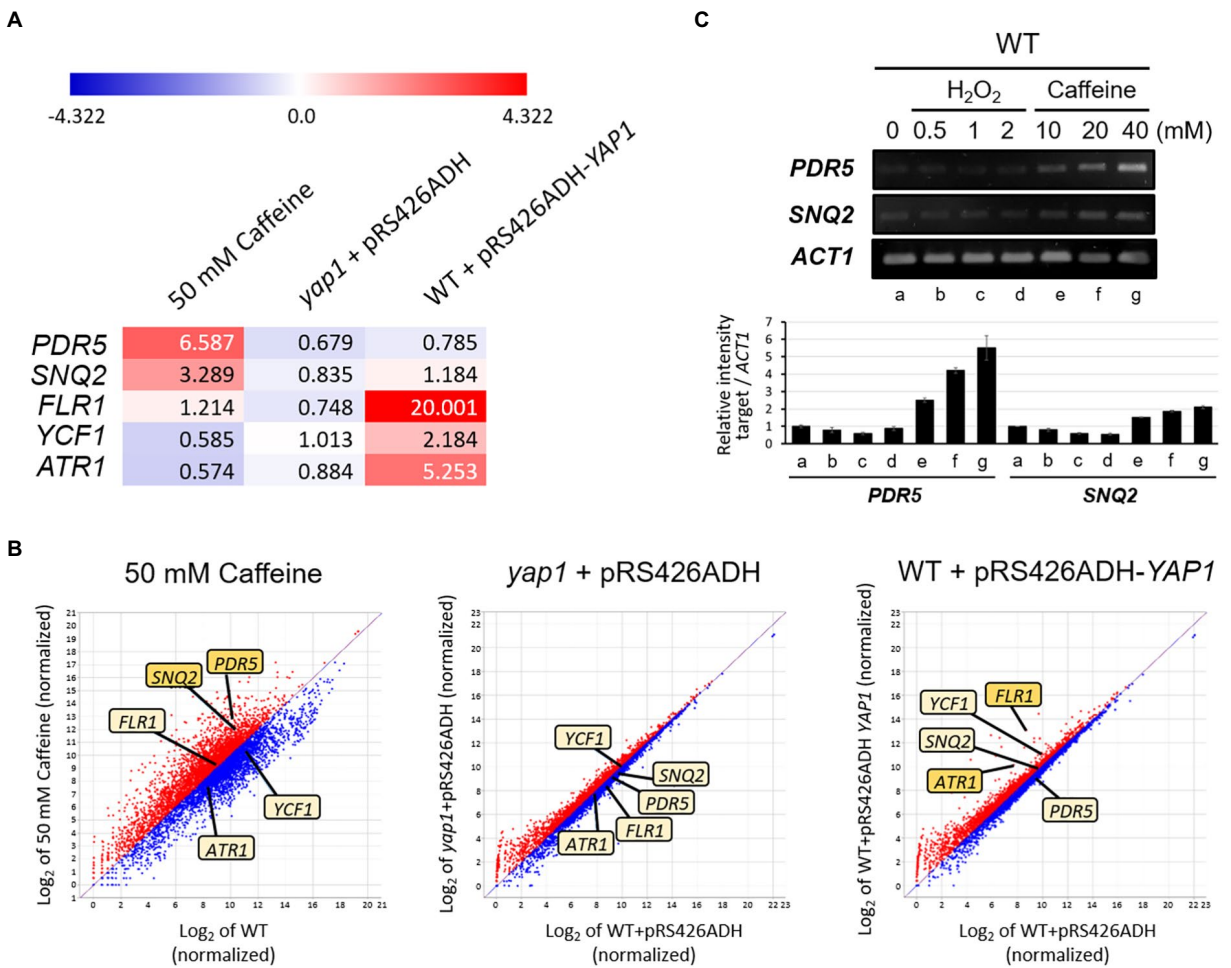
QuantSeq analysis to screen Yap1-dependent target genes for caffeine resistance. **(A)** Heat map depiction with numerical values of induction and **(B)** Scatter plot show differentially expressed mRNA levels of several transporter genes in caffeine-treated WT, *yap1* and *YAP1* overexpression strains. Red color represents upregulation and blue represents downregulation of mRNA expression. The degrees of darkness in colors represent the proportions of the expressive level. The 7,125 mRNAs were standardized and log2-transformed to show on a scatter plot by ExDEGA software. The X-axis displays the expression level of genes from WT, whereas the Y-axis shows the expression level of genes **(C)** RT-PCR analysis of *PDR5* and *SNQ2* expression in cells with H_2_O_2_ or caffeine treatment. Cells were incubated at 30°C for 30 min with H_2_O_2_ or caffeine treatment. The numbers of PCR cycles used are all 28.

To more thoroughly screen for unknown genes whose expression is induced by caffeine, and to investigate whether the induction is involved in the activation of Yap1, we performed quantitative analysis of transcriptomes in three groups of strains: 50 mM caffeine-treated WT, *yap1* mutant, and Yap1 overexpressed cells (Supplementary Figure 1). Out of 7,125 genes that were analyzed, the transcription of 2,285 genes (32%) was upregulated with a fold change of > 1.5 by 50 mM caffeine. This number is about three times higher than that in the *yap1* mutant (9%) or Yap1 overexpression strain (11%). Among these genes, transcription levels of at least seven encoding multidrug transporters that were implicated in pleiotropic drug resistance, including *PDR5* and *SNQ2*, were upregulated by over 1.5-fold after 1 h of treatment with 50 mM caffeine, and the expression of *PDR5* was two times higher than that of *SNQ2* (Figures 5A,B; Supplementary Figure 2). This result is consistent with a previous report showing that Pdr5 and Snq2 are two major caffeine efflux pumps in *S. cerevisiae* (Tsujimoto et al., 2015). Consistent with transcriptome results, we also found that in quantitative PCR analysis transcriptional induction of *PDR5* is higher than that of *SNQ2* by various concentrations of caffeine, but not by H_2_O_2_ (Figure 5C).

However, the transcription of *FLR1*, *YCF1*, and *ATR1* did not increase in response to caffeine. Instead, mRNA levels of these three genes were elevated under the condition of Yap1 overexpression, as expected for Yap1-dependent genes, and this upregulation disappeared completely in the absence of Yap1 (Figure 5A). Specifically, the expression of *FLR1* increases significantly up to 20-fold, the highest among transporter-encoding genes, and *ATR1* with over 5-fold increase is the next. These results are consistent with our previous observation that the caffeine-resistant phenotype of Yap1 overexpression strain completely disappeared in an *flr1* mutant background and was slightly impaired in an *atr1* mutant (Figure 2B).

### Reactive oxygen species signaling modulates cellular adaptation to caffeine in a Yap1-dependent manner

**Figure 6:**
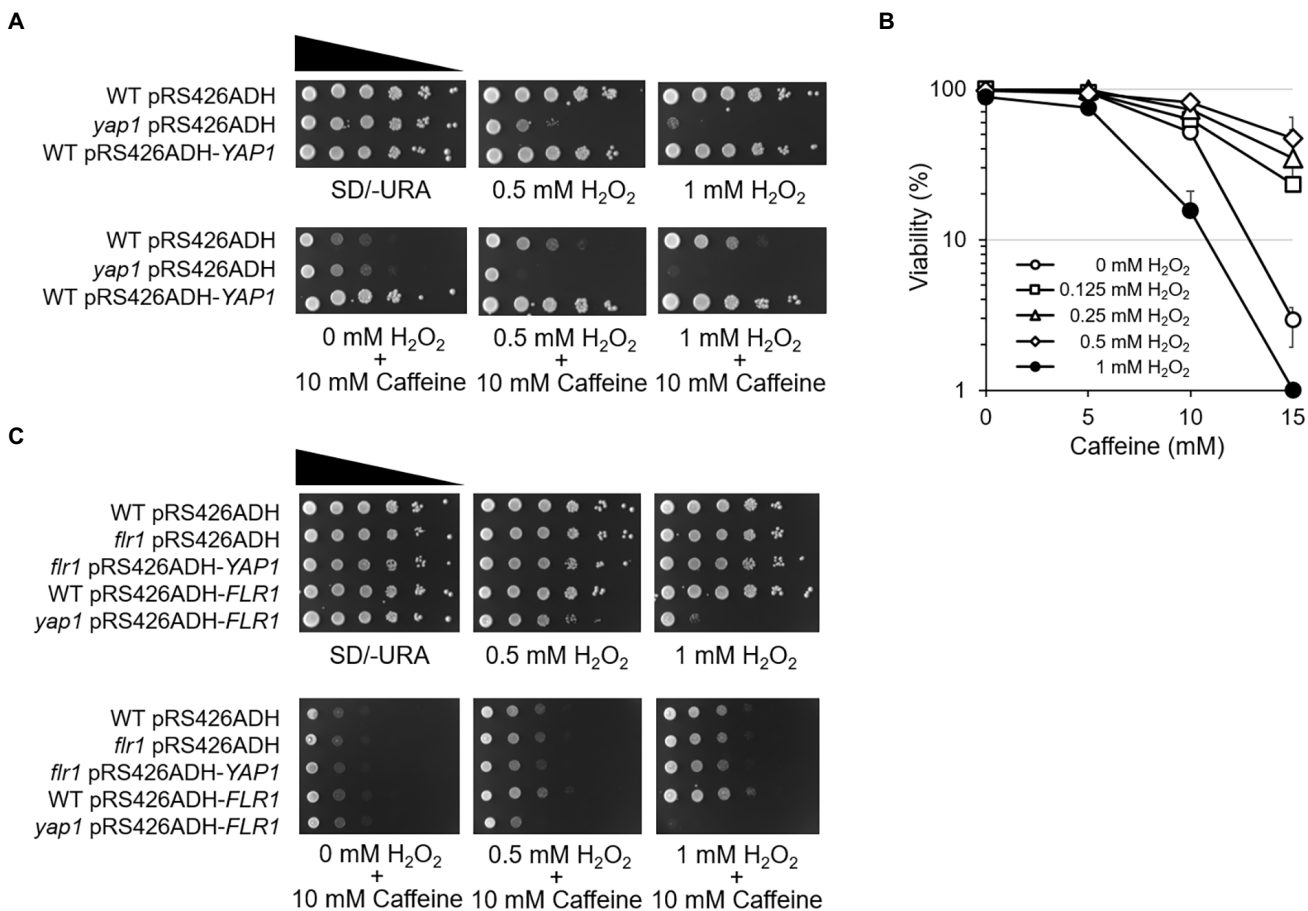
ROS signaling modulates cellular adaptation to caffeine. **(A)** 10-fold serial dilutions of yeast mutant strains were spotted on SD/-URA media in the presence of caffeine with or without H_2_O_2_ and incubated for 3 days at 30°C. **(B)** Cell viability in the presence of caffeine was measured by counting CFUs on YEPD plates with or without H_2_O_2_. **(C)** 10-fold serial dilutions of yeast mutant strains were spotted on SD/-URA media in the presence of the caffeine with or without H_2_O_2_ and incubated for 3 days at 30°C.

An observation that the cellular process to acquire caffeine tolerance involves the activation of the oxidative stress response pathway aroused our curiosity as to whether ROS signaling modulates cellular adaptation to caffeine toxicity. We examined the caffeine sensitivity of cells with or without oxidative stress by using spotting analysis. While cell growth is severely impaired on minimal media with 10 mM caffeine as shown earlier in Figure 1B, the impaired growth of WT cells is significantly restored when cells are grown on caffeine media and treated with 0.5 mM H_2_O_2_, and even more with 1 mM H_2_O_2_ (Figure 6A). However, the recovery of caffeine resistance completely disappeared in the absence of Yap1. These results give a strong implication that the oxidative stress response is mediated by Yap1 in adaptation to caffeine toxicity. The concentration of H_2_O_2_ used for this analysis is not sufficient to inhibit cell growth solely, but enough to generate caffeine tolerance when treated together with caffeine.

To consolidate the notion that ROS signaling renders cell adaptation to caffeine, cell viability was analyzed using a CFU counting assay. When 100 of WT cells were spread onto 15 mM caffeine-containing plates, only ~ 2% were viable (Figure 6B). Interestingly, the viability increases as higher concentrations of H_2_O_2_ are treated with caffeine, and reached up to ~ 60% with 0.5 mM H_2_O_2_. However, cells are no longer viable with 1 mM H_2_O_2_ on plates containing 15 mM caffeine.

Next, spotting analysis was carried out with *flr1* mutants and Flr1 overexpression strains to address whether Flr1 is involved in ROS-mediated caffeine adaptation. Surprisingly, we found that H_2_O_2_-induced caffeine adaptation still occurs in the absence of *FLR1* (Figure 6C). Yap1 overexpression does not make *flr1* mutants more resistant to caffeine when treated with H_2_O_2_ compared to WT or *flr1* mutant cells, although *flr1* mutant survives caffeine toxicity better with H_2_O_2_ than without H_2_O_2_. However, *yap1* mutants display no adaptation even with Flr1 overexpression. These results suggest that Yap1, but not Flr1, is essential for oxidative stress-mediated cellular adaptation to caffeine toxicity.

### Reciprocal crosstalk between ROS signaling and caffeine resistance

Caffeine has potential antioxidant activity, protecting cells against oxygen radicals and repairing some oxidative damages (Devasagayam et al., 1996; Vieira et al., 2020). Since several toxic drugs are known to trigger ROS production in the cell and many transporters responsible for multidrug resistance belong to oxidative stress-responsive regulons, we next investigated the possible roles of caffeine in the regulation of oxidative stress and the accompanying DNA damages and mutagenesis (Chung, 2021).

**Figure 7:**
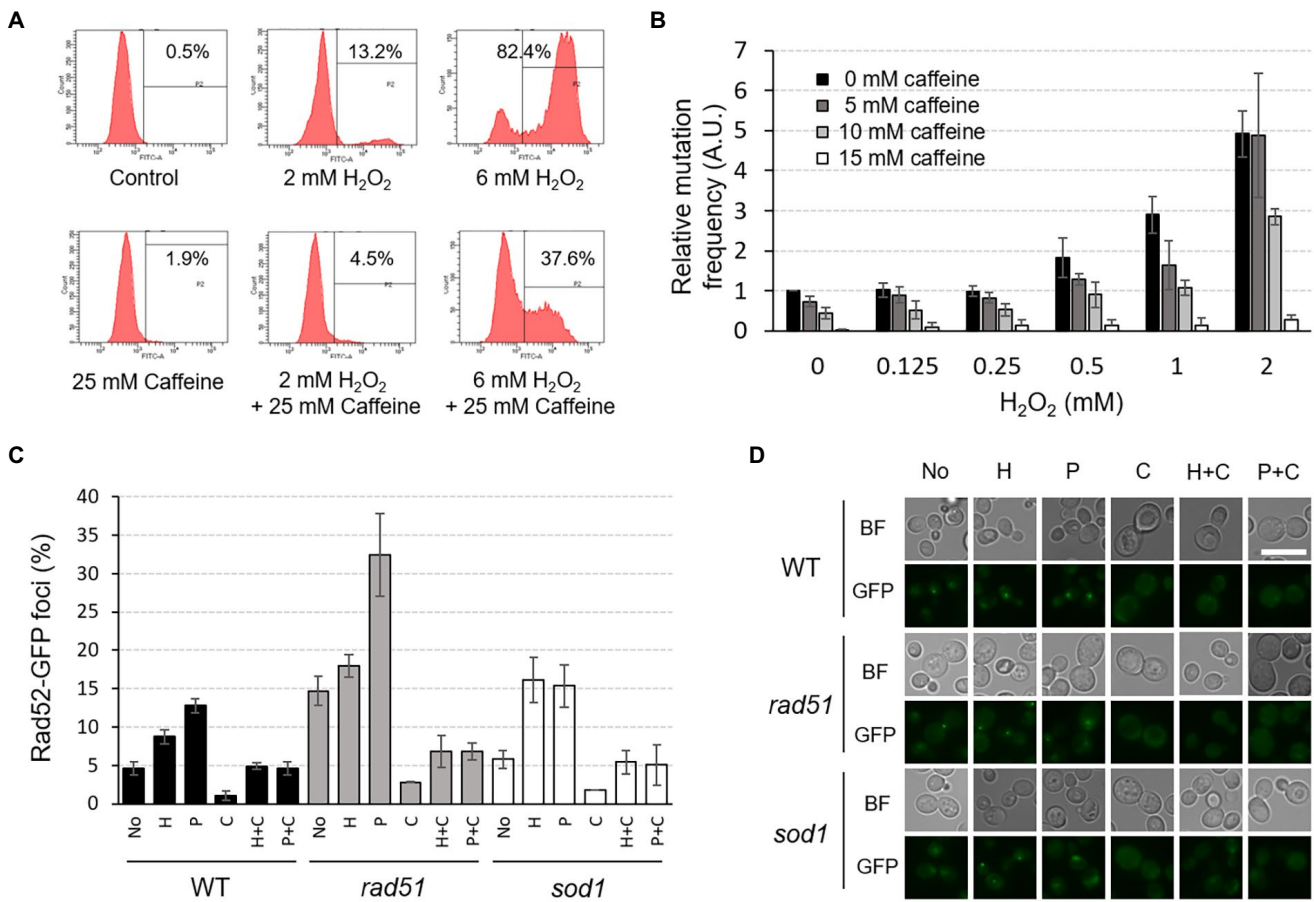
Caffeine reduces intracellular ROS level, mutation rates, and Rad52-GFP foci formation induced by H_2_O_2_ or phleomycin treatment. **(A)** The intracellular ROS level was measured by FACS analysis under the indicated conditions. Cells were treated with H_2_O_2_ and/or caffeine for 2 h prior to ROS detection. P2 percentage indicates the proportion of cells with an increased production of ROS, emitting above the background levels of DCF fluorescence. Averages from three independent experiments are presented. **(B)** Relative mutation frequency was measured by *CAN1* forward-mutation assay in response to various concentrations of H_2_O_2_ with or without caffeine treatment. **(C)** The proportion of cells containing Rad52-GFP foci was determined under the indicated conditions of H_2_O_2_ (H), phleomycin (P), and caffeine (C) in WT, *rad51*, and *sod1* mutant strains. **(D)** Fluorescent microscopy of Rad52-GFP foci analyzed in (C). White scale bar corresponds to 10 μm. BF, bright field.

When the intracellular ROS level was measured using flow cytometry with H_2_DCFDA, a cell-permeant indicator for general oxidative stress, we found that the amount of ROS in WT cells increases significantly when treated with higher concentrations of H_2_O_2_ (Figure 7A). However, co-treatment of 25 mM caffeine with 6 mM H_2_O_2_ dramatically reduces ROS levels from 82.4 to 37.6%, although caffeine on its own shows almost no change in ROS level (1.9%).

To test whether caffeine directly affects genome stability, we measured spontaneous mutation frequency using *CAN1* forward-mutation assay with or without H_2_O_2_ and caffeine. Consistent with our previous observations, mutation rates of WT cells start to increase from 0.5 mM concentration of H_2_O_2_ and reached up to five times at 2 mM H_2_O_2_ treatment compared to those with no treatment (Yi et al., 2016; Figure 7B). However, when cells are treated with caffeine together with H_2_O_2_, the mutation rates decrease in a concentration-dependent manner, and the rate with 15 mM caffeine is about 100-fold lower than that of the untreated WT. The increased mutation rate with 2 mM H_2_O_2_ decreased significantly to ~ 95% with 15 mM caffeine compared with no caffeine treatment. Caffeine seems to remove ROS and successfully mask its harmful effects on mutagenesis.

The number of Rad52-GFP foci in the nucleus, an indicator of accumulated DNA damage including DSBs, also decreases significantly when cells are treated with caffeine (Figures 7C,D). The foci formation induced by H_2_O_2_ or phleomycin is inhibited efficiently when treated together with caffeine, and similar results appeared in cells lacking Rad51 or Sod1, showing increased intracellular DSBs or ROS, respectively. All these observations reveal that caffeine acts as a strong antioxidant and protects cells from potential genome instability and mutagenesis by inhibiting the generation of DNA lesions.

**Figure 8:**
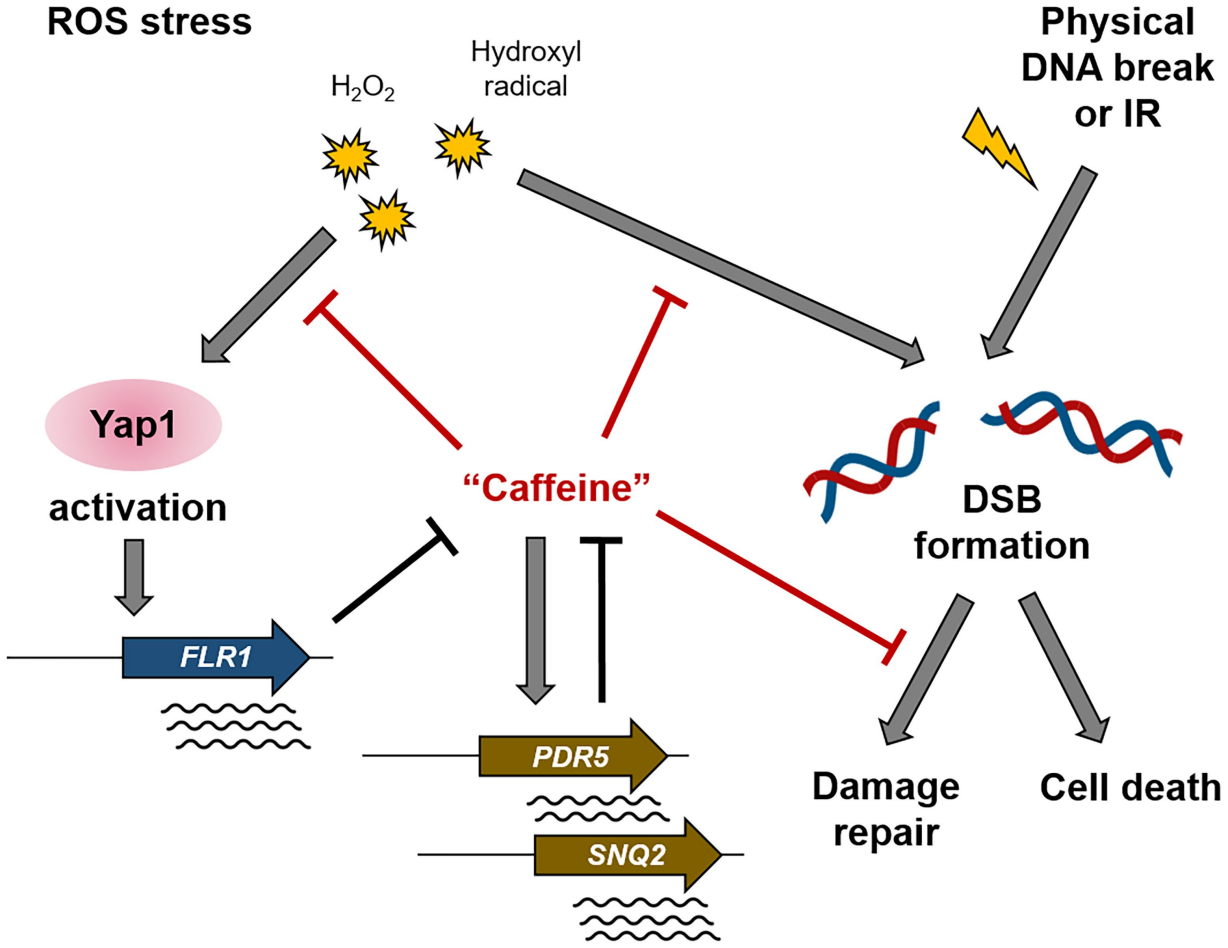
A schematic diagram illustrating the mechanism of action of caffeine in Yap1-dependent ROS signaling and DNA damage response. The ROS-induced Yap1 activation protects cells from caffeine toxicity, and caffeine reduces intracellular ROS level, DNA damage, and mutation rates. Controversially, caffeine disturbs several steps of DSB repair pathway.

Taken together, our results coherently indicate reciprocal crosstalk between oxidative stress response and caffeine metabolism (Figure 8). Yap1 activation by elevated ROS levels and the subsequent induction of its downstream target genes, undoubtedly including Flr1, confer cells resistance to caffeine toxicity, and inversely, appropriate doses of caffeine play important roles in the removal of deleterious oxidants from cells and the protection of chromosomes from ROS-mediated damage and accompanying mutagenesis.

## Discussion

Caffeine elicits well-documented cytotoxic effects to bacteria and various eukaryotic cells including yeast and humans, inactivating a lot of important cellular activities. Caffeine abolishes DNA damage-induced cell cycle arrest by inhibiting DNA damage response (DDR) signaling pathway orchestrated by the sensor kinases, ATM and ATR, and perturbs multiple steps in DNA damage repair pathways (Blasina et al., 1999; Sarkaria et al., 1999; Tsabar et al., 2015b). In this study, however, we addressed several possible beneficial roles of caffeine that maintains chromosomal fidelity by reducing intracellular ROS level and inhibiting DSB formation and mutagenesis. We showed that Yap1 and its target gene *FLR1* are the key mediators of regulatory crosstalk between ROS signaling and cellular resistance to caffeine.

Although the export of a wide range of structurally and functionally unrelated drugs out of the cell by a single transporter is still open to debate, our results demonstrate that Flr1, previously known as an efflux pump of fluconazole, diazaborine, and methotrexate, is also highly involved in caffeine detoxification. The exact mechanism of its involvement in caffeine tolerance is not clarified, but the process might be related to the regulation of physiological conditions such as cellular redox state or metal concentrations since Yap1, a highly conserved transcription factor for oxidative stress response and cadmium resistance, is required for the induction of *FLR1* expression (Wemmie et al., 1994; Alarco et al., 1997; Kuge et al., 2001; Figure 2).

Flr1 is a yeast representative of the major facilitator superfamily (MFS) of multidrug resistance. The promoter region of *FLR1* contains three Yap1-responsive elements (YREs), each with differential binding activity to Yap1 (Nguyên et al., 2001). Therefore, the transcription of *FLR1* is induced by oxidizing agents such as H_2_O_2_, diamide, diethylmaleate, and even the alkylating agent methyl methanesulfonate (MMS), which stalls replication forks leading to DNA DSBs.

In fact, a number of oxidative stress regulons are responsible for the expression of the multidrug resistance system including ABC transporters. A total of 22 genes encode ABC transporters in the *S. cerevisiae* genome and many of them are crucial for adaptation to environmental stresses (Paumi et al., 2009). Among them, two major caffeine efflux pumps, Pdr5 and Snq2, and a Yap1-dependent cadmium transporter, Ycf1, have been reported to be commonly involved in both multidrug resistance and the oxidative stress response (Buechel and Pinkett, 2020). Miyahara et al. (1996) have shown that both *PDR5* and *SNQ2* have one YRE sequence in their promoter regions. This is plausible because many toxic drugs could trigger ROS accumulation in the cell.

However, our QuantSeq analysis and several studies with mutants revealed that the expression of neither *PDR5* nor *SNQ2* seems to be regulated by Yap1, and *FLR1* is the only strong target of Yap1 activation among the potential candidates responsible for caffeine resistance (Figures 2, 5). In *S. cerevisiae*, 9 proteins, including Pdr5, and Snq2, have been identified as pleiotropic drug resistance (PDR) protein subfamily members, but Flr1 does not belong to that subfamily (Prasad and Goffeau, 2012). It is possible that the transcriptional induction of *PDR5* and *SNQ2* is also dependent on Yap2, a functional homolog of Yap1 with overlaps in oxidative and metal stress response, but with gene targets that are distinct from Yap1, especially during oxidative stress (Stephen et al., 1995; Buechel and Pinkett, 2020).

The reason why Pap1-impaired *S. pombe* cells are sensitive to caffeine is that the expression of the efflux pump Hba2 is significantly reduced in the absence of Pap1 (Calvo et al., 2009). Excessive production of ABC transporter Caf5 also enhances caffeine resistance in a Pap1-dependent manner. What can be the functional counterparts of Hba2 or Caf5 in *S. cerevisiae*? We presented several RT-PCR and QuantSeq data showing that the transcription of *FLR1*, *ATR1*, and *YCF1* is clearly induced by Yap1, but none of them are actually required for caffeine resistance (Figures 2, 5A). Overexpression of *FLR1* fails to confer cells with as much resistance to caffeine as overexpression of Yap1 (Figures 2B, 3B). Most importantly, however, overexpression of Yap1 shows an obvious caffeine resistant effect only in the presence of Flr1, indicating that other unknown Yap1 target proteins could work for caffeine tolerance together with Flr1 (Figure 2B). Interestingly, overexpression of Yap1 also confers resistance to cerulenin, a potent inhibitor of fatty acid synthase through the Flr1 transporter, and this observation again demonstrates that the Yap1-Flr1 stress response system has a wide spectrum of detoxifying capability (Oskouian and Saba, 1999).

It has been reported that the *QDR3*, encoding another member of multidrug transporters in the MFS, is induced by 20 mM caffeine treatment and is also crucial for cellular resistance to cisplatin and bleomycin, DNA DSB-inducing reagents (Tenreiro et al., 2005; Kuranda et al., 2006). High-throughput microscopic screening by Tkach et al. (2012) showed that the level of spontaneous Rad52 foci is elevated in *atr1* null mutant. These observations suggest that the efficacy of caffeine efflux pumps is also highly involved in the protection or repair of deleterious DNA damages as well as protection against oxidative stress.

In this research, we provided strong evidence that a relatively moderate concentration of caffeine plays a role as an antioxidant, scavenging harmful ROS out of the cell, and pretreatment with H_2_O_2_ could provide adaptation to the cytotoxic effects of caffeine in a Yap1-dependent manner (Figure 6). Importantly, while Yap1 overexpression-mediated caffeine resistance required *FLR1*, H_2_O_2_-induced cellular adaptation to caffeine toxicity relied on the presence of Yap1, but not *FLR1* (Figures 2, 6). This implies that ROS-induced Yap1 activation could be somehow different from the overexpression of Yap1 in the transcriptional induction of Yap1 target genes, or it is probably because increased H_2_O_2_ might alter broader range of transcriptional profiles involved in caffeine tolerance than just a Yap1-related group of genes.

Consistent with the recent report by Czachor et al. (2020) that coffee infusions reduce Rad52-GFP foci formation almost by half, our results demonstrated that caffeine itself is effective in decreasing both DNA DSB damages and spontaneously occurring mutations (Figure 7). Caffeine inhibits the repair of damaged DNA by disturbing the correct operation of repair proteins and damage checkpoint signaling, but at the same time, caffeine attenuates cytotoxic effects by efficiently reducing intracellular ROS level in the earlier steps as well as damage-induced mutagenesis (Saiardi et al., 2005; Tsabar et al., 2015a, 2015b; Figure 8). Taken together, we conclude that caffeine regulates a variety of cellular metabolic processes as a double-edged sword; cells maintain well-balanced growth condition by reciprocal crosstalk between the effects of caffeine and the ROS signaling pathway, especially in a Yap1-dependent manner.

## Data availability statement

The data presented in the study are deposited in the NCBI GEO repository, accession number GSE214902.

## Author contributions

JC designed and performed almost all experiments. S-HH performed a part of experiments. W-HC designed the entire research and wrote the manuscript. All authors contributed to the article and approved the submitted version.

## Funding

This work was supported by Priority Research Centers Program through the National Research Foundation of Korea (NRF) funded by the Ministry of Education (NRF-2020R1A2C1011370 and 2016R1A6A1A03007648).

## Conflict of interest

The authors declare that the research was conducted in the absence of any commercial or financial relationships that could be construed as a potential conflict of interest.

## Publisher’s note

All claims expressed in this article are solely those of the authors and do not necessarily represent those of their affiliated organizations, or those of the publisher, the editors and the reviewers. Any product that may be evaluated in this article, or claim that may be made by its manufacturer, is not guaranteed or endorsed by the publisher.

## Supplementary material

The Supplementary material for this article can be found online at: https://www.frontiersin.org/articles/10.3389/fmicb.2022.1026780/full#supplementary-material

## Footnotes

^1^ http://david.abcc.ncifcrf.gov/

^2^ http://www.ncbi.nlm.nih.gov/

^3^ https://www.ncbi.nlm.nih.gov/geo/query/acc.cgi?acc=GSE214902

## Abbreviations

WT, Wild type
ROS: Reactive oxygen species
4-NQO: 4-Nitroquinoline-N-oxide
GFP: Green fluorescent protein
H_2_DCFDA: 2′,7′-dichlorodihydrofluorescein diacetate
TOR: Target of rapamycin
HR: Homologous recombination
DSB: Double-strand break
ATM: Ataxia-telangiectasia mutated
ATR: ATM-related
PDR: Pleiotropic drug resistance
ABC transporter: ATP-binding cassette transporter
CFU: Colony forming unit
