# Supplementary material for: Yap1-mediated Flr1 expression reveals crosstalk between oxidative stress signaling and caffeine resistance in Saccharomyces cerevisiae
Source: Front Microbiol. 2022 Nov 23;13:1026780. doi: 10.3389/fmicb.2022.1026780 (PMC9726721; doi:10.3389/fmicb.2022.1026780)
Supplement: Supplementary file 1 [file Data_Sheet_1.docx]

### Supplementary information

Strains used in this study are listed in Supplementary Table 1.

**SupplementaryTable 1. Strain list**

| Strain name | Parental strain | Genotype | Reference |
| --- | --- | --- | --- |
| BY4741 | - | *MAT****a*** *his3*Δ1 *leu2*Δ0 *lys2*Δ0 *ura3*Δ0 |  |
| BY4742 | - | *MAT***α** *his3*Δ1 *leu2*Δ0 *lys2*Δ0 *ura3*Δ0 |  |
| yWH537  yWH1457  yWH1456  yWH1671  yWH1458  yWH1509 | BY4741  BY4741  BY4741  BY4742  BY4741  BY4741 | WT  *yap1::KANMX*  *skn7::KANMX*  *sod1::HIS3*  *tsa1::KANMX*  WT pRS426ADH | this study  this study  this study  this study  this study  this study |
| yWH1521  yWH1582  yWH1515  yWH1665  yWH2382  yWH2383 | BY4741  BY4741  BY4741  BY4741  BY4741  BY4741 | WT pRS426ADH*-YAP1-TAP*  WT pRS426ADH-*SKN7-TAP*  WT pRS426ADH-*SOD1-TAP*  WT pRS426ADH-*TSA1-TAP*  *pdr5::KANMX* pRS426ADH  *pdr5::KANMX* pRS426ADH-*YAP1-TAP* | this study  this study  this study  this study  this study  this study |
| yWH2384  yWH2385  yWH2464  yWH2465  yWH2488  yWH2489  yWH2490  yWH2491  yWH2509  yWH2510  yWH2533  yWH2546  yWH2568  yWH2576  yWH1510  yWH2572  yWH2580  yWH1464  yWH1463  yWH1579  yWH1885 | BY4741  BY4741  BY4741  BY4741  BY4741  BY4741  BY4741  BY4741  BY4741  BY4741  BY4741  BY4741  BY4741  BY4741  BY4741  BY4741  BY4741  BY4741  BY4741  BY4741  BY4741 | *snq2::KANMX* pRS426ADH  *snq2::KANMX* pRS426ADH-*YAP1-TAP*  *pdr5::KANMX snq2::NATMX* pRS426ADH  *pdr5::KANMX snq2::NATMX* pRS426ADH-*YAP1-TAP*  *flr1::KANMX* pRS426ADH  *flr1::KANMX* pRS426ADH-*YAP1-TAP*  *ycf1::KANMX* pRS426ADH  *ycf1::KANMX* pRS426ADH-*YAP1-TAP*  *atr1::KANMX* pRS426ADH  *atr1::KANMX* pRS426ADH-*YAP1-TAP*  *pdr5::KANMX snq2::NATMX*  *pdr5::HIS3MX snq2::NATMX flr1::KANMX*  WT pRS426ADH*-FLR1*  *flr1::KANMX* pRS426ADH-*FLR1*  *yap1::KANMX* pRS426ADH  *yap1::KANMX* pRS426ADH-*FLR1*  *pdr5::KANMX snq2::NATMX* pRS426ADH*-FLR1*  WT *YAP1-GFP-HIS3*  WT *RAD52-GFP-HIS3*  *rad51::LEU2 RAD52-GFP-HIS3*  *sod1::KANMX RAD52-GFP-HIS3* | this study  this study  this study  this study  this study  this study  this study  this study  this study  this study  this study  this study  this study  this study  this study  this study  this study  this study  this study  this study  this study |

Primer sets used in this study are listed in Supplementary Table 2.

**SupplementaryTable 2. Primer sets**

| Primer name | Usage | Sequence | Reference |
| --- | --- | --- | --- |
| FLR1-RT-F1 | RT-PCR | CCGTTTTTGCAGGCAATGGTTT (22 mer) | This study |
| FLR1-RT-R1 | RT-PCR | TGTACGTAATGATGGCCCATAC (22 mer) | This study |
| SOD1-RT-F1 | RT-PCR | GAACGTGGGTTCCACATTCATG (22 mer) | This study |
| SOD1-RT-R1 | RT-PCR | CCACACCATTTTCGTCCGTCTT (22 mer) | This study |
| ACT1-RT-F1 | RT-PCR | TCTGCCGGTATTGACCAAACTA (22 mer) | This study |
| ACT1-RT-R1 | RT-PCR | TCATGGAAGATGGAGCCAAAGC (22 mer) | This study |
| PDR5-RT-F1 | RT-PCR | TTTTCTAGTGCCGCCTGGGTTA (22 mer) | This study |
| PDR5-RT-R1 | RT-PCR | CACCTGGGTTTAGGCAACCATC (22 mer) | This study |
| SNQ2-RT-F1 | RT-PCR | AACCGCGTTGACTGATCCAAAT (22 mer) | This study |
| SNQ2-RT-R1 | RT-PCR | TCTCTTGAGCCATCGATTCGTC (22 mer) | This study |
