# Supplementary figures and images for: Yap1-mediated Flr1 expression reveals crosstalk between oxidative stress signaling and caffeine resistance in Saccharomyces cerevisiae
Source: Front Microbiol. 2022 Nov 23;13:1026780. doi: 10.3389/fmicb.2022.1026780 (PMC9726721; doi:10.3389/fmicb.2022.1026780)

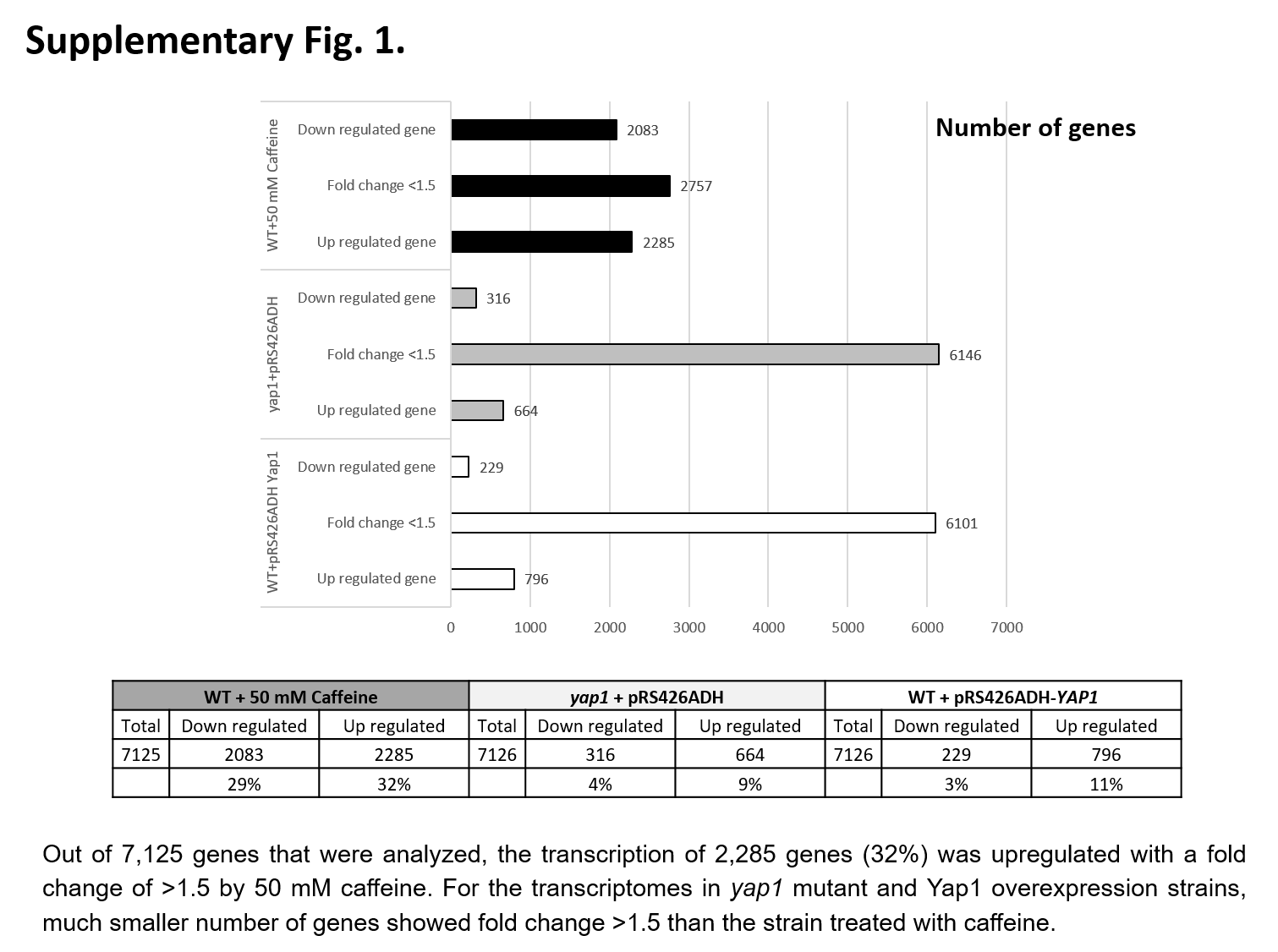

Supplement: Supplementary file 2 [file Image_1.TIF]

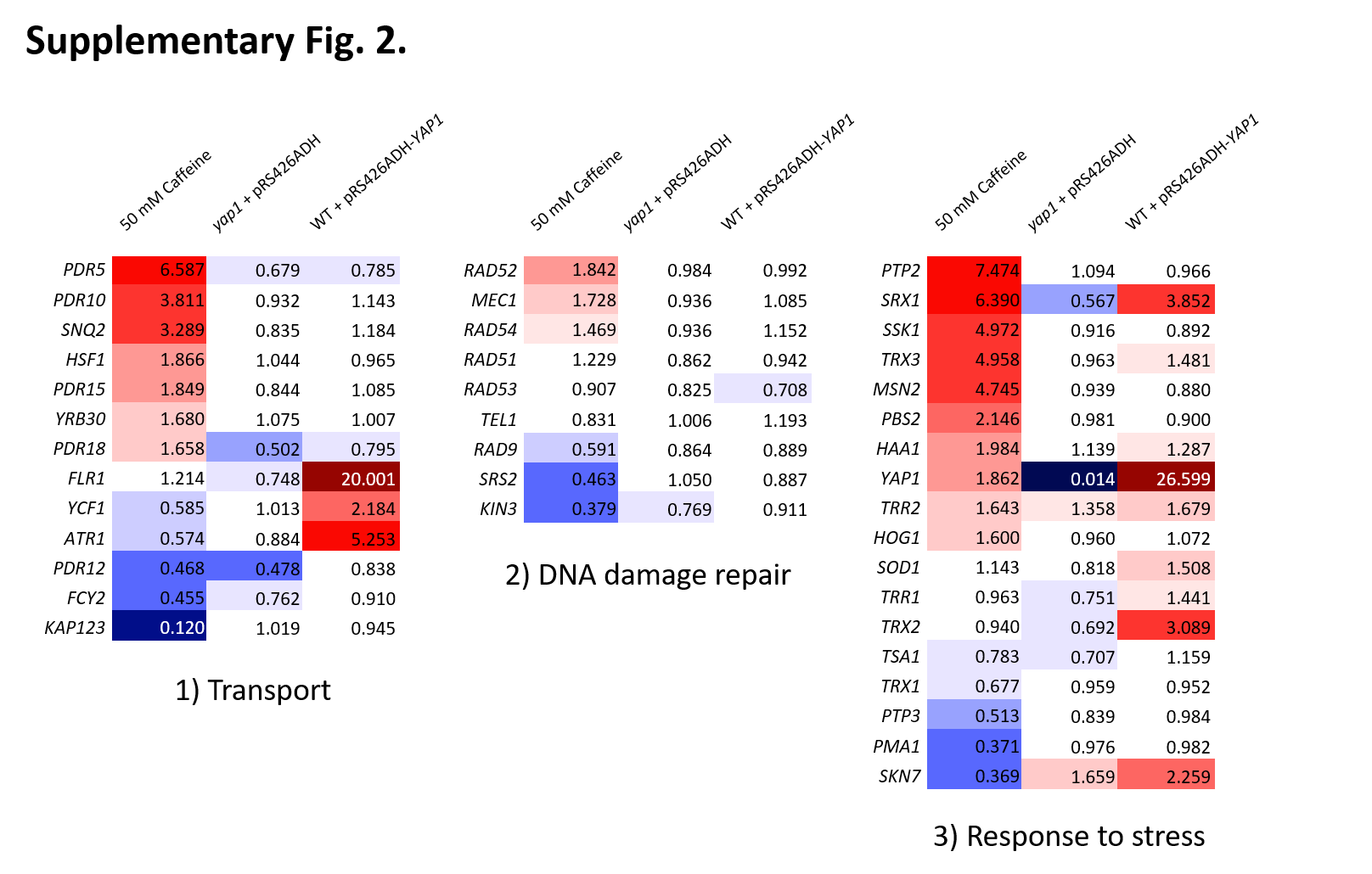

Supplement: Supplementary file 3 [file Image_2.TIF]

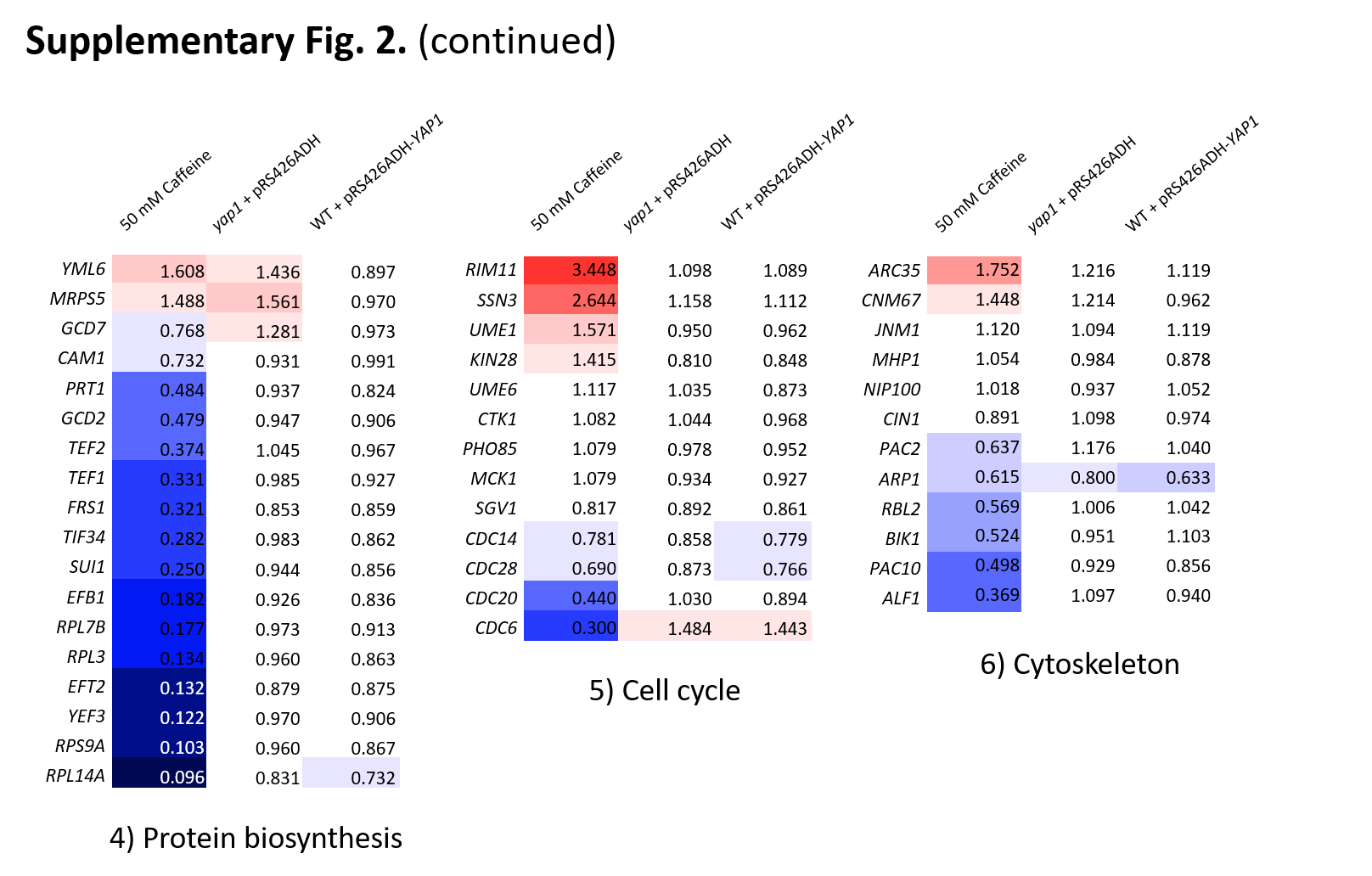

Supplement: Supplementary file 4 [file Image_3.TIF]

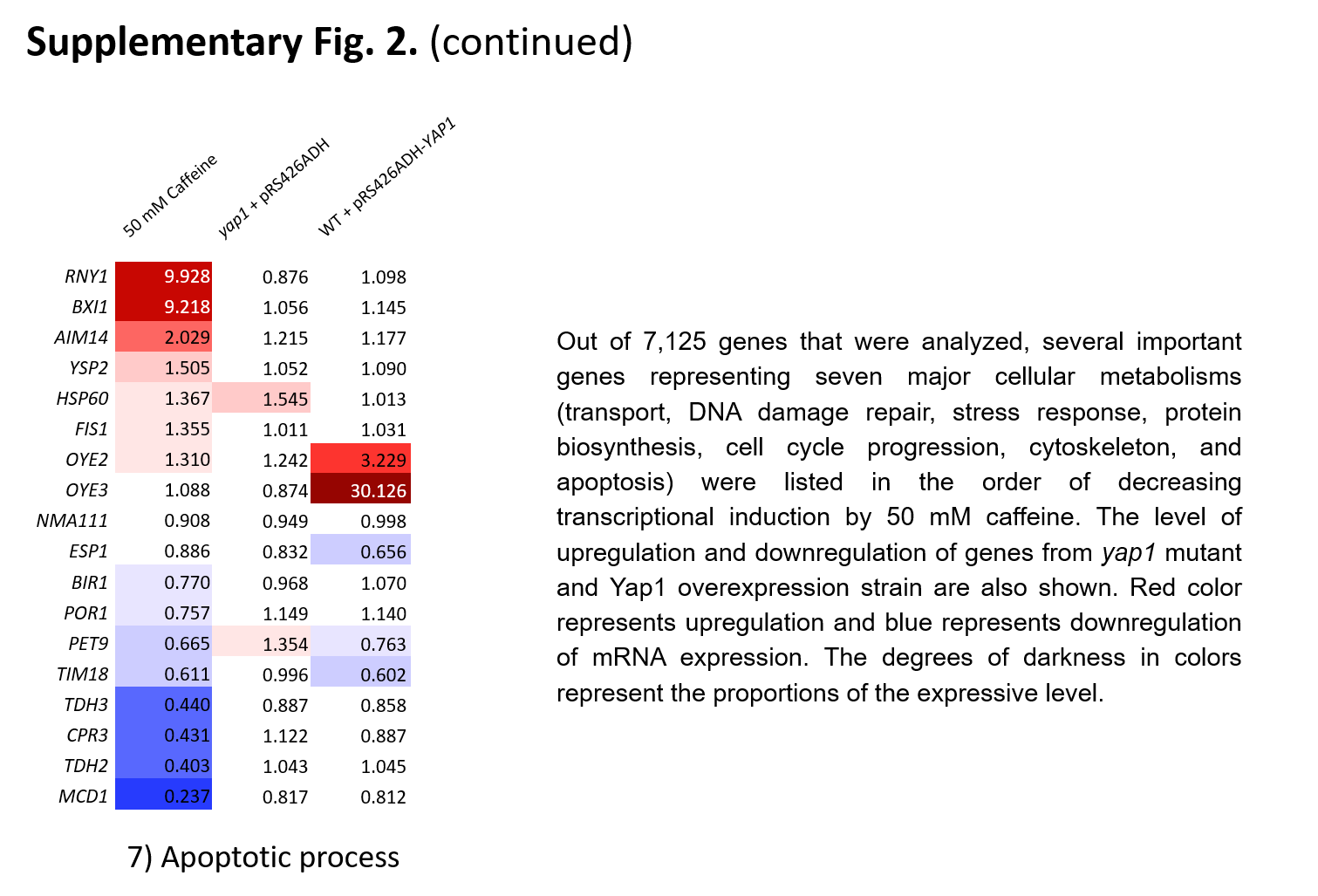

Supplement: Supplementary file 5 [file Image_4.TIF]
